# The Ali Krogius procedure for treatment of patellofemoral instability should be regarded as obsolete even in skeletally immature patients

**DOI:** 10.1186/s12891-022-05200-4

**Published:** 2022-03-16

**Authors:** Yannic Bangert, Felix Mittelstrass, Johannes Weisshorn, Sébastien Hagmann, Alexander Barié, Ayham Jaber

**Affiliations:** 1 Department of Orthopedics, Center for Orthopedics, Trauma Surgery and Spinal Cord Injury, Heidelberg University Hospital, Schlierbacher Landstrasse 200a, 69118 Heidelberg, Germany; 2 Department of Diagnostic and interventional Radiology, Aachen University Hospital, Pauwelsstraße 30, 52074 Aachen, Germany; 3 Center for Joint Surgery and Sport Injuries, Sportopaedie Heidelberg, Clinic St. Elisabeth Heidelberg, Max-Reger-Straße 5-7, 69121 Heidelberg, Germany

**Keywords:** Ali krogius, Patellar instability, Pediatric, patella, Knee, Patellar dislocation, Mpfl

## Abstract

**Background:**

Several interventions are established for treating patellofemoral instability in adults. Fewer exist for pediatric patients without damaging the epiphysis. The Ali Krogius (AK) method is currently still being used. Most studies are not current and report varying results in small patient population. The aim of this study is to determine the long-term results of the AK method.

**Methods:**

In this monocentric, retrospective study design, 33 knees in 33 patients who received the AK procedure for recurrent patellar dislocation were assessed. The average age was 20.8 years (range 6–40). The following functional scores were assessed: Kujala Score, Lysholm Score and Tegner Score. Subgroup analysis was done for patients ≤16 years of age. Available preoperative imaging was assessed for known risk factors.

**Results:**

After an average follow-up of 7.8 years (Range 59–145 months), a total of 8 (24%) knees suffered a redislocation postoperatively. Seven of the eight dislocations occurred in patients ≤ 16 years of age. One knee (3%) was revised due to persistent pain. The median score was 86 points for the Kujala score and 90 for the Lysholm score. The median in the Tegner score was level 6. Clinically, the patellar glide was lateralized in 7 knees (21%) and an apprehension sign was triggered in 8 knees (24%).

**Conclusions:**

Including the present study, the existing literature indicates a redislocation rate between 24 and 41% following AK. It should thus be regarded as obsolete even though it protects the epiphysis. Surgical interventions such as medial patellofemoral ligament reconstruction with femoral drilling distal to the epiphysis should be preferred.

**Trial registration:**

Retrospectively registered: S-302/2016.

**Level of evidence:**

III

## Background

Patellar dislocation is common in children and adolescents. The incidence is reported to be around 43 per 100,000 in patients younger than 16 years of age [1]. The etiology is diverse. Two forms can be distinguished. The chronic patellar instability due to a congenital malformation of the patellar stabilizers and the chronic, secondary patellar instability after traumatic patellar dislocation [2]. The first etiology is significantly more common and occurs more frequently in female patients [3]. Several anatomical and functional factors contribute to the recurrent dislocations. These include trochlear dysplasia, patella alta, lateral patellar tilt, increased tibial extra-torsion, increased femoral anteversion, vastus medialis hypoplasia, subtalar joint pronation or valgus deformity of the lower limb [4]. A variety of different surgical treatment options exist depending on the severity and etiology. The goal is proximal and/or distal realignments to achieve a stable patella [5].

The Elmslie-Trillat procedure is indicated in patellofemoral instability related to a pathological Tuberositas-Tibiae-Trochlea-Groove-Index (TTTG-Index) and involves medialization of the ligamentum patellae with the corresponding part of the tibial tubercule [6]. Trochleoplasty is an invasive procedure to deepen the trochlear groove indicated in patients with patellar instability due to a “C” or “D” and in some cases “B” trochlea according to Dejour [7]. The Roux–Goldthwait procedure was initially described by Roux in 1888 and then modifed by Goldthwait in 1895 as hemi-patellar transfer for the treatment of recurrent patellar dislocation [8]. The Ali Krogius (AK) procedure was first described in 1904 for the treatment of patellofemoral instability [9]. A strip of the medial retinaculum, pedicled proximally into the vastus medialis, is sewn into the lateral retinaculum This results in a proximal active and passive medialization restraint of the patella. The AK procedure is a purely soft tissue operation and offers the advantage of protecting open growth plates. Unfortunately, studies from the 1980s report a rather high rate of dislocation [10, 11]. The procedure is still practiced in many institutions and is mentioned as a standard procedure in book references for orthopaedic surgery. Current studies do not exist. The aim of the present study is to report results of the Ali Krogius procedure in a considerable number of patients with a long-term follow-up in order to provide a current statement and recommendation regarding its use.

## Methods

**Fig. 1:**
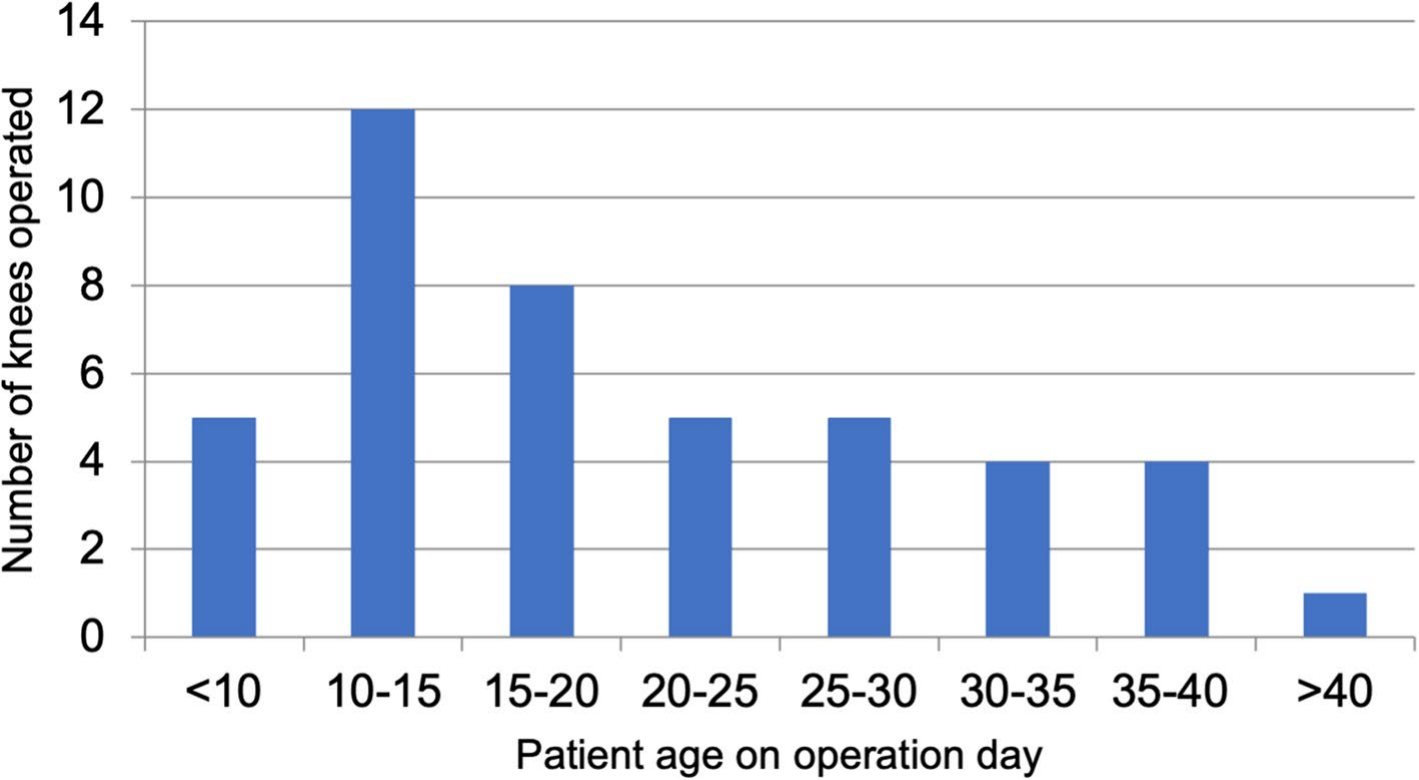
Age distributions of the study population

The present retrospective monocentric cohort study investigates all patients with recurrent patellar dislocation (3 or more instances of patellar dislocation) treated with the AK surgical method in one university hospital between January 1, 2004 and December 31, 2011. During this period, 93 knees in 81 patients received the procedure and were included. Patients with an underlying neurological disease or congenital syndromes (32 patients) that predispose to patellar dislocation were excluded in order to provide a more reliable conclusion. Patients who received other procedures (i.e. Roux–Goldthwait / Galeazzi / femoral or tibial osteotomies) in combination with AK were also omitted (11 patients). As a result, 54 knees were excluded from the study. A total of 39 knees in 38 patients remained in the cohort. Five patients were lost to follow-up. Thirty-three knees in thirty-three patients (70% female, 30% male) were therefore included in the final cohort. The patients’ age at the time of surgery ranged from 6 to 40 years, with an average age of 20.8 years. Age distribution is demonstrated in Fig. 1. Seventeen of the thirty-three examined knees belonged to patients who were 16 years old or younger at the time of surgery.

A clinical examination was carried out with an average duration of 94 months (Range 59–145 months). Relevant examination parameters such as the Zohlen sign (patellar grind test), apprehension sign and J-sign were performed. Range of motion (ROM) of the knee was reported [12]. Scores relevant to patellofemoral instability, pain and function which have been tested for validity and reliability in the literature were assessed [13, 14]. These included the Kujala Anterior knee pain scale, Lysholm Score, IKDC-2000, Knee Injury and Osteoarthritis Outcome Score (KOOS) and Tegner activity Score [15–19]. A subgroup analysis was done in patients aged ≤16 years and those older. Postoperative redislocations and revisions were reported. Preoperative radiological evaluation of available radiographs and Magnetic Resonance Imaging (MRI) was done in patients when available. The Caton-Deschamps index was used to evaluate patella alta (Caton-Deschamps Index > 1,2) [20]. High-grade trochlear dysplasia was defined as Dejour types B, C, and D [21].

Data acquisition and analysis were performed in compliance with protocols approved by the Ethical Committee of the corresponding medical faculty (S-302/2016). The study was registered in the German Register of Clinical Studies and was conducted in accordance with the Declaration of Helsinki. All Patients gave informed written consent to participate in the study.

Descriptive statistics were used to report frequencies and means for the cohort and subgroups. A 2-tailed t-test was performed to compare data. Statistical analysis was performed using SPSS version 26. *P*-value < 0.05 was considered statistically significant.

### Surgical method

The procedure was performed under general anesthesia. The patient was placed supine on the operating table. A tourniquet was applied to the thigh, the leg exsanguinated, and the cuff inflated to 250 mmHg.

**Fig. 2:**
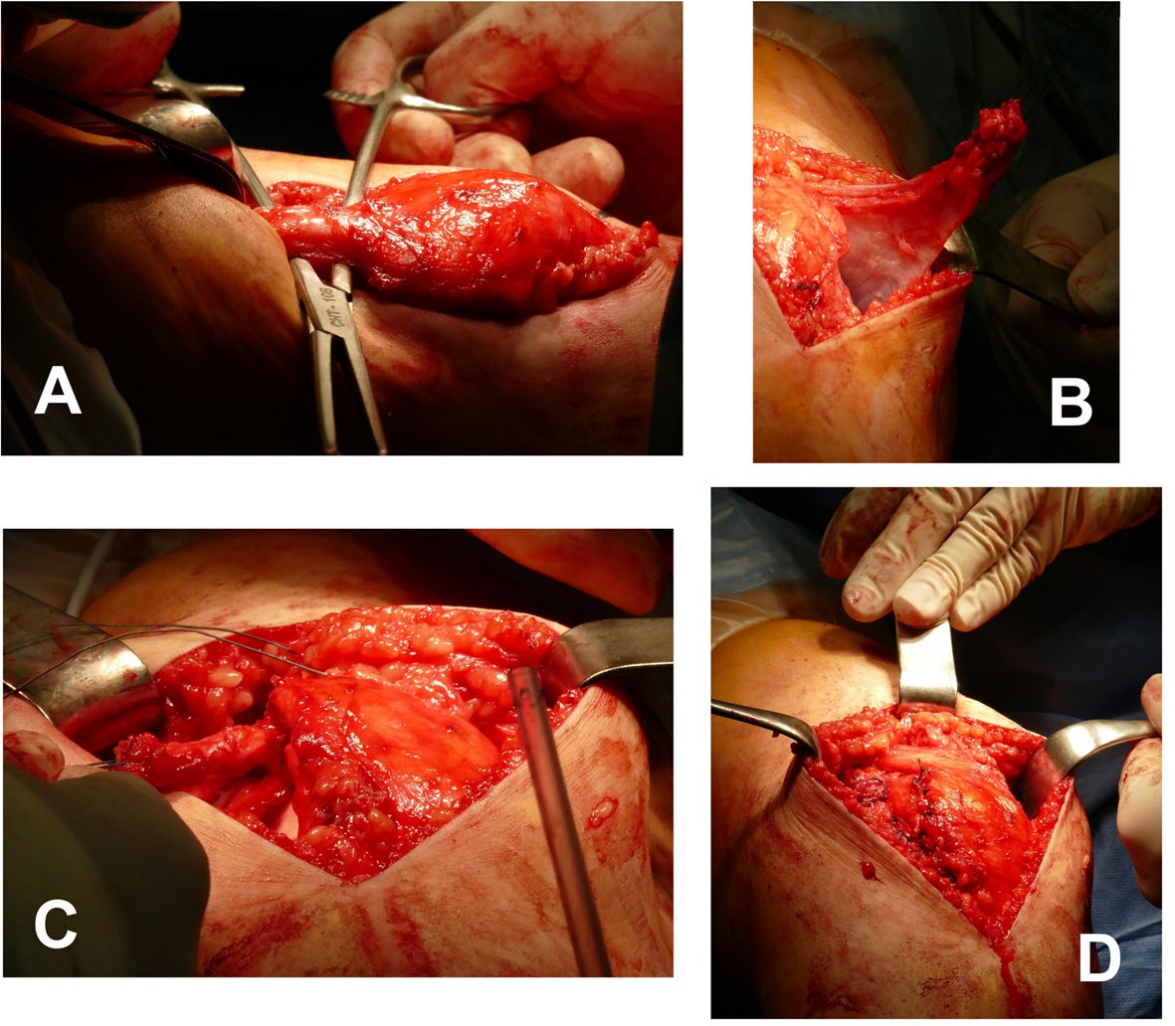
Intraoperative demonstration of the Ali Krogius method. **A**: Undermining the Quadriceps. **B**: Pedunculated retinaculum strip. **C**: Lateralization of the retinacular strip. **D**: Fixed, medialized patella after the Ali Krogius procedure

**Fig. 3:**
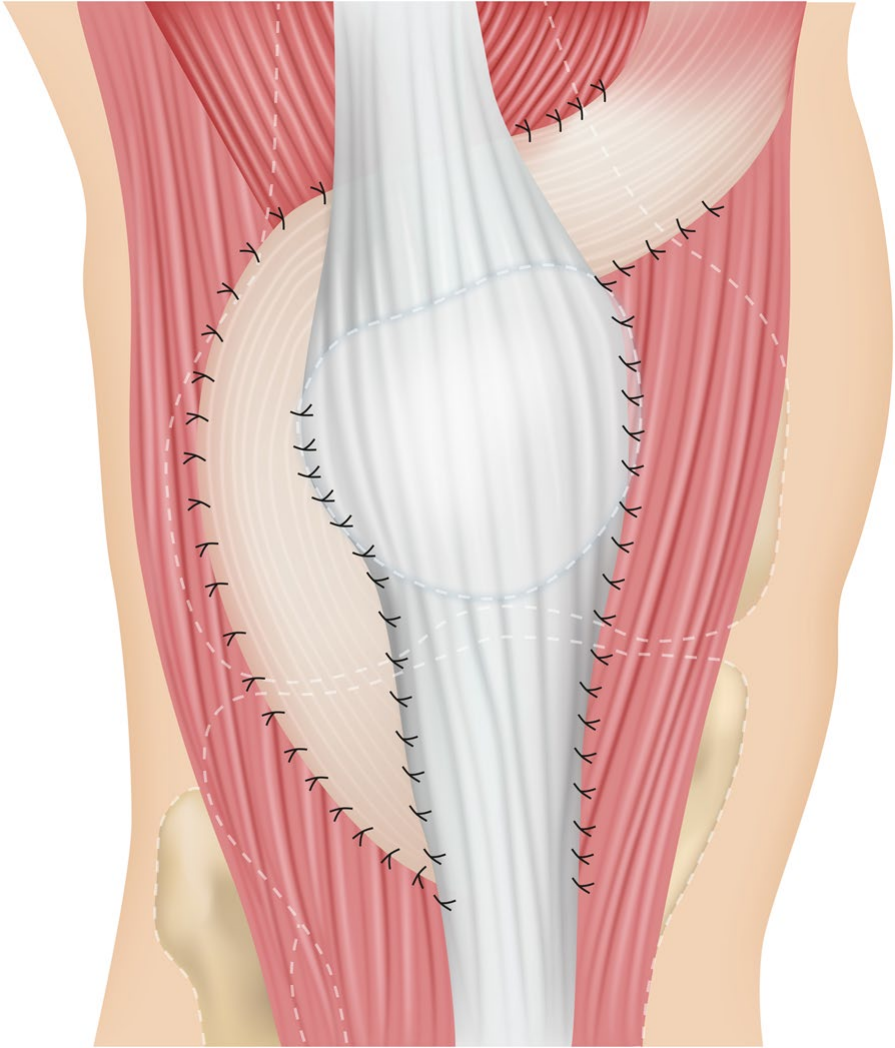
Illustration of the Ali Krogius surgical method

A skin incision running along the middle of the patella is made reaching the tibial tuberosity. The medial and lateral retinaculum are first exposed. The medial retinaculum is split lengthways approximately 1 cm medial to the patella and 2–3 cm proximal to the tibial tuberosity in order to prepare a proximally pedicled capsular retinaculum strip approximately 1 cm wide, depending on the degree of the desired correction. Care is taken to ensure that the retinaculum strip is sufficiently stalked into the vastus medialis muscles to ensure that the proximal medialization is as uniform as possible. This capsule-retinacular strip is then thread-reinforced distally and, with slight tension, repositioned laterally under the quadriceps tendon and anchored to the lateral retinaculum margins. The medial retinaculum is now adapted by means of a single button suture and with constant monitoring of the patellar path. The degree of medialization can be increased at any time by overlapping suturing of the medial retinaculum margins. The method is demonstrated in Figs. 2 and 3.

All patients received regular physiotherapy postoperatively. A 2-week duration of partial weightbearing with approximately 20 kg was instructed initially instructed. This was followed by a 2-week duration of gradual build up to full weightbearing. The flexion of the operated knee joint did not exceed 90 ° degrees for 6 weeks. A rigid frame brace was worn for a total of 3 months postoperatively with the above-mentioned flexion restriction for the first 6 weeks. Cycling and swimming were allowed 6 weeks postoperatively, jogging after 12 weeks and full fitness after muscle strengthening 6 months postoperatively.

## Results

The preoperative radiological examination was possible in 26 of 33 knees. Fifteen of twenty-six knees (58%) had a Caton-Deschamps index > 1.2 and thus a patella alta. A patella tilt angle > 5 ° was measured in 19 of 26 knees (73%). MRI revealed a type A trochlea in 12 patients, type B in 2 patients, type C in 2 patients and type D in 6 patients.

**Fig. 4:**
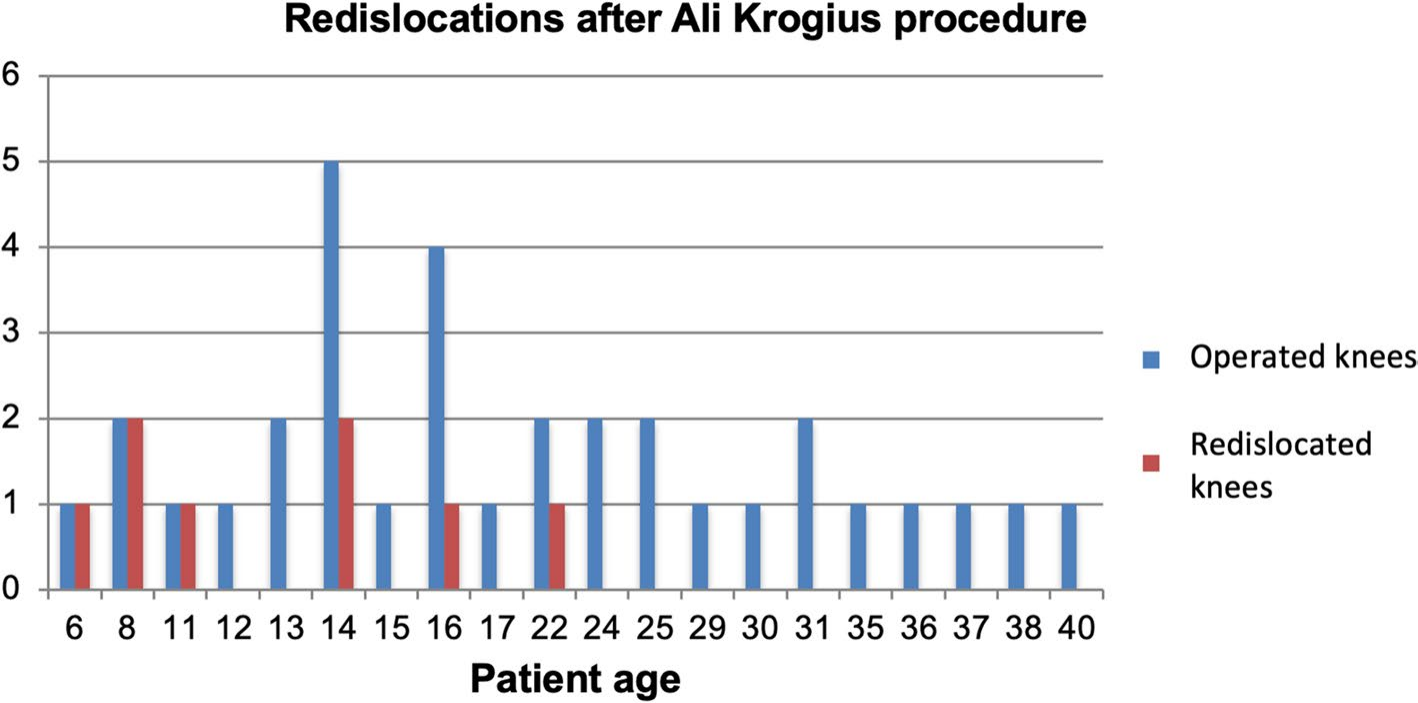
Redislocations after Ali Krogius procedure according to patients’ age

In the 33 knees that received AK, 8 knees (24.2%) suffered a redislocation. The patients’ age at the time of dislocation was ≤16 years in 7 of the 8 cases. Stratified according to patient age, the patient group aged 16 years or younger therefore has a significantly higher rate of dislocation of 41% (Fig. 4) than those over 16 years of age (6%). Preoperative imaging was available for 5 of the 8 redislocated knees. Two patients (40%) had a patella alta. A high grade trochelar dysplasia (Type B and D) was detected in two patients (40%). The remaining patients had a mild grade trochlear dysplasia (Type A). Despite redislocations, these patients showed good to very good clinical results. The median Kujala score was 81%, the Lysholm score 82%, the IKDC-2000 86%, the KOOS score 90% and the Tegner score a median of 5.

On physical examination on follow-up, knee flexion range had a median of 140 ° (range 110 °-150 °) and extension range of 5 ° (Range 0 ° -20 °). In comparison, the non-operated knees showed a flexion range of 140 ° (range 120 ° -150 °) and extension range of 5 ° (range 0 ° -20 °). When comparing the ROM of the operated and non-operated knee joints using the T-test, there were no significant differences in flexion (*P* = 0.45) and extension (*P* = 0.34). The J-sign was positive in 3 knees. One of these knees was dislocated postoperatively. The apprehension sign could be triggered in 8 of the 33 knees. In 3 of these knees, the patella redislocated postoperatively. Of all patients, 13 (39%) had a valgus deformity, 3 (9%) a varus deformity and 17 (51.5%) a straight leg axis. In addition, 7 (21%) had a lateralized patellar glide and 26 (79%) had a centered patellar glide.

**Fig. 5:**
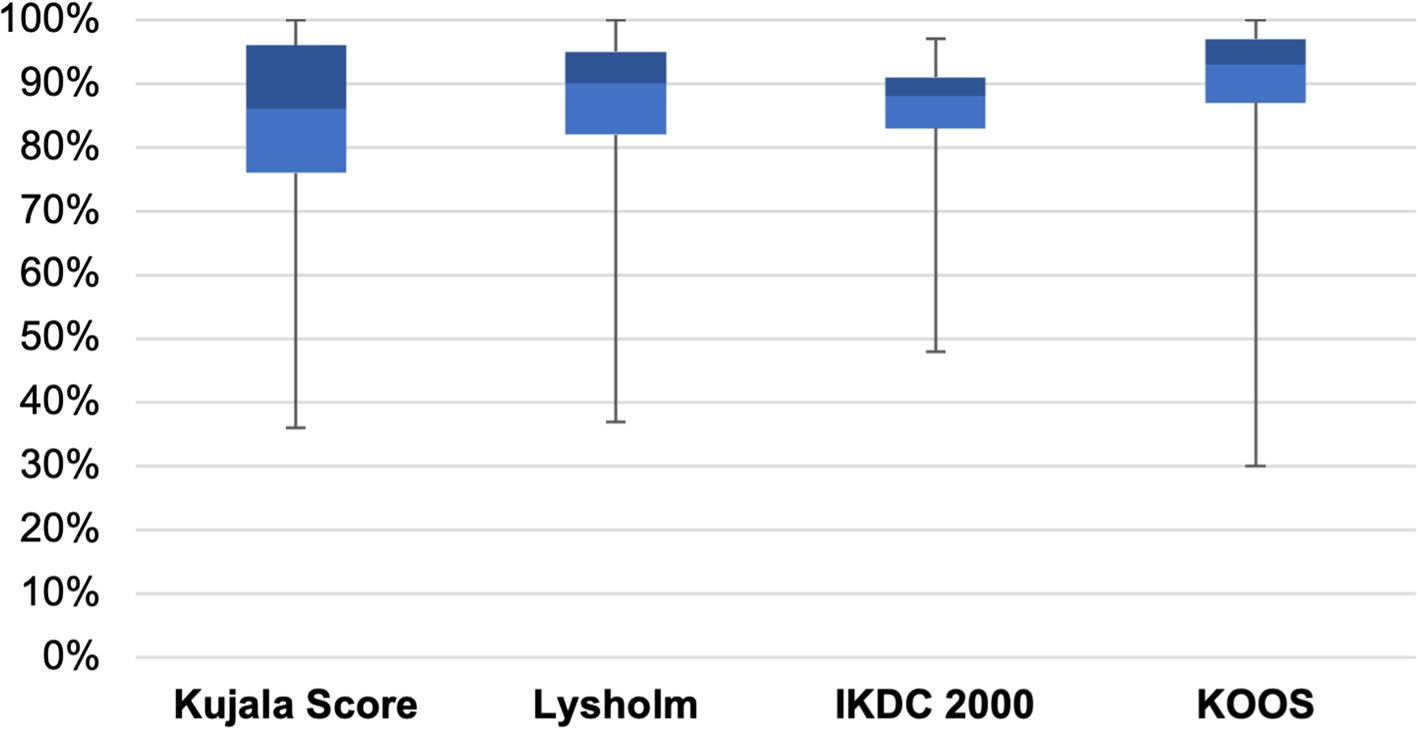
Results of the score on follow-up

The postoperative scores showed good to very good results (Fig. 5). The median was 86% in the Kujala score, 90% in the Lysholm score, 88% in the IKDC 2000, 93% in the KOOS score and 6 in the Tegner score. Eleven patients (33.3%) achieved a very good result in the Lysholm score, 13 (39.4%) patients a good result, 8 patients (24.2%) a sufficient result and 1 patient (3%) a bad result. In the IKDC-2000, 30 patients (91%) reported results in the range of the reference values and 3 patients (9%) reported results below the reference values [22]. In the Tegner activity score, both men and women had a median of 6.

**Fig. 6:**
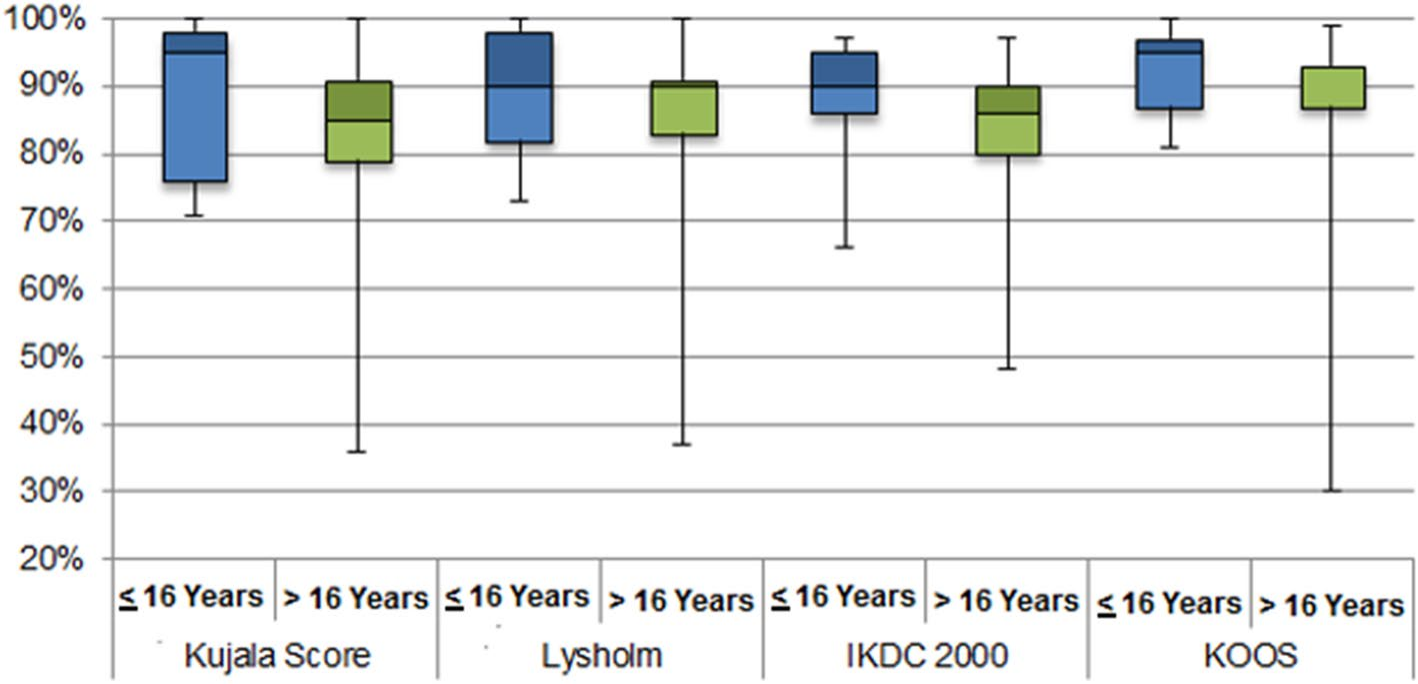
Subgroup analysis of patients according to age group

Subgroup analysis were performed considering patients age of **≤ **16 years and >  16 years indicating approximate time of closed physis. The available preoperative values of both subgroups are displayed in Table 1. Statistical comparison of the available values revealed no significant differences. Regarding the follow-up assessment, a mean of 5.3 showed a significantly higher value in the Tegner Score (*P* = 0.03) for those over 16 years. The Kujala score (*P* = 0.097), Lysholm score (*P* = 0.15), IKDC 2000 score (*P* = 0.089) and KOOS score (*P* = 0.085) showed higher scores among those aged 16 years or younger (Fig. 6), but statistical comparison was not significant.

**Table 1:** Preoperative clinical and radiological values with ranges where applicable

	Total Number	Average age	Leg Alignment	Trochlear Dysplasia (Dejour)	Caton-deschamps ratio	Patellar Tilt	Kujala score
**> 16 years**	16	29 (17–40)	5 Valgum 9 Normal 1 Varus	5 Type A 2 Type B 1 Type C 3 Type D	1.27 (0.85–1.66)	11° (2–29)	53% (35–73%)
**≤ 16 years**	17	13 (6–16)	7 Valgum 7 Normal 3 Varus	7 Type A 1 Type C 3 Type D	1.30 (0.86–1.65)	10° (3–26)	57% (41–83%)

## Discussion

There are many different conservative and surgical procedures that address different predisposing risk factors for the treatment of recurrent patellar dislocation. No gold standard exists for the surgical treatment. Many of the procedures that have become established in recent years, such as Elmslie’s tuberosity medialization or trochleaplasty, are bone-invasive and are contraindicated in young patients due to open growth plates [23, 24]. Medial-based soft-tissue stabilization procedures are the focus in this pediatric population. A method for which there are only a few studies with varying results in the medical literature is the AK procedure performed as described in the methods section.

In the present study, a total of 33 knee joints received the AK procedure. In comparison with other studies, the number of knees operated on is roughly as large as that of published studies with 24–34 operated knee joints and smaller than 2 other studies with 73 and 49 operated knee joints (Table 2) ([9, 11, 25–28]. The average age of the operated patients was 20.78 (range 6–40), which is consistent with other studies on the AK method and also other epidemiological studies in which an average age of 20–22 years is reported [9, 25, 28–31]. The follow-up period in the present study was more than twice as long as that in the studies by Petrick and Tischer and Bauer et al. selected follow-up period of 2.8 years and slightly longer than that of Rebmann et al. and Jalovaara et al. with follow-up durations of 6.5 and 7 years, respectively [9, 11, 25, 27, 28]. To date, no study has been published on the results of the AK method with a follow-up period longer than 7.7 years.

**Table 2:** Existing literature including the present study reporting results following the AK procedure

Authors	Year	Follow-up in Months	Number of knees	Redislocation rate
Cotta H.	1959	?	27	1 (4%)
Petrick P. et al.	1978	34	31	4 (13%)
Bauer FC. et al.	1984	34	34	14 (41%)
Rebmann K. et al.	1989	78	49	18 (36%)
Jalovaara P. et al.	1988	84	73	31 (42%)
Heisel J. et al.	1983	?	24	6 (25%)
Present study	2021	94	33	8 (24%)

When comparing the ROM of the operated and non-operated knee joints using the T-test, no significant differences in the ROM were found in this study. 13 (39%) patients had a valgus deformity. The proportion of patients who also have a genu valgum with recurrent patellar dislocation is therefore significantly smaller compared to the 77% described in a cross-sectional study [31]. Whether the valgus deformity is predisposing to a recurrent patellar dislocation cannot be definitively proven based on these figures. No comparative values regarding the clinical parameters such as J-sign and apprehension sign exist.

At 24%, the rate of redislocations in the present study is lower than that of Bauer et al., Jalovaara et al. and Rebmann et al., who describe a rate of 36–42% and roughly comparable to that of Heisel et al., with a redislocation rate of 25% [9, 11, 25–28]. The revision rates after AK in the studies by Heisel et al. (12.5%), Jalovaara et al. (7%) and Bauer et al. (23.5%) are higher than in the present study (3%) [25, 27, 28].

In skeletally immature patients, a conservative therapy regimen is initially recommended if the first dislocation of the patella is uncomplicated. After exhaustion of conservative therapy and persistence of the symptoms in children with recurrent patellar dislocation, surgical treatment must be considered. This is especially the case if massive bone deformities or traumatic osteochondral injuries are involved [32]. In skeletally immature patients, bony transpositions should primarily be discouraged, and growth-respecting surgical stabilization techniques such as the AK procedure, preferred [23].

The identification of the medial patellofemoral ligament (MPFL) as the most important passive stabilizer in the extended knee has led to considerable progress in understanding patellar dislocation. Patients of growing age have more frequent injuries to the MPFL than adults with an injury rate of up to 99% following the first dislocation [33]. MPFL reconstruction yields a high success with excellent clinical results and low redislocation rates postoperatively (5–7%) [34–36]. A meta-analysis comparing MPFL reconstruction with other soft tissue realignment techniques in skeletally immature patients reported a statistically insignificant difference in instability rate, It was evident, however, that MPFL reconstruction had superior results [37]. Techniques using the adductor magnus tendon as a “sling” for the femoral fixation minimize risk to the distal femoral physis. However, the attachment site of the reconstruction on the femur nonanatomic and is too proximal, potentially resulting in a non-isometric graft, loss of knee motion, and extension of the graft as growth occurs [38]. Considering these risks, multiple physeal-sparing MPFL reconstruction techniques have emerged which aim to prevent injury to the physis and at the same time adequately augment the MPFL [39, 40]. These techniques produced good preliminary results, but more studies are required to establish them further.

### Strengths and limitations

The validity of this study could have been enhanced by control groups and a prospective study design. One aspect worth mentioning is the size of the study population. Since the AK method is only rarely used, this number of cases is relatively high compared to other studies on the results of this surgical method. Only in the retrospective cohort studies by Jalovaara and Rebmann in the 1980s were similarly large case numbers examined. Moreover, the subgroup analysis assessed patients with a cut-off at 16 years of age as a comparison of adult and pediatric populations. However, this does not necessarily separate patients with and without epiphyseal closure.

In comparison with the other retrospective studies on the AK method, the average follow-up period of 94 months (7.8 years) is comparatively long. However, the range is quite large with 59–145 months. In addition, due to the long-time interval between the date of the operation and the follow-up examination, only 85% of the patients who had met the study criteria took part in the study. The remaining 15% could no longer be contacted due changing addresses or names. Their redislocation rates remain unknown. Since a period of several years was examined, the patients in the study collective were treated by different surgeons, which limits comparability. In follow-up studies, a prospective study design should be used in which pre- and postoperative score collection and imaging are compared over time to present the subjective and objective postoperative results more precisely. In the present study, a preoperative radiological assessment was unfortunately not possible for all patients.

## Conclusions

Even if the protection of the growth plate speaks for the use of the AK procedure in skeletally immature patients, it must be described as obsolete due to the relatively high rate of redislocation. The present study retaliates an important message which is consistent with older existing studies in the medical literature. Other surgical interventions such as MPFL reconstruction with femoral drilling distal to the growth plate should thus be preferred.

## Data Availability

The datasets used and/or analysed during the current study are available from the corresponding author on reasonable request.

## Abbreviations

AK: Ali krogius
MPFL: Medial patellofemoral ligament
TTTG-Index: Tuberositas-Tibiae-Trochlea-Groove-Index
ROM: Range of motion
KOOS: Knee Injury and Osteoarthritis Outcome Score
MRI: Magnetic resonance imaging
i.e.: Id est
